# “I gave birth, and then I disappeared”—women's experience of primary care follow-up and lifestyle prevention after gestational diabetes

**DOI:** 10.1080/17482631.2026.2717466

**Published:** 2026-08-15

**Authors:** Amanda Björk Javanshiri, Sara Modig, Peter Nymberg, Susanna Calling

**Affiliations:** a Center for Primary Health Care Research, Department of Clinical Sciences, Malmö, Lund University, Malmö, Sweden; b University Clinic Primary Care Skåne, Skåne University Hospital, Region Skåne, Sweden

**Keywords:** Gestational diabetes mellitus, type 2 diabetes mellitus, qualitative research, semi-structured interviews, primary health care, health promotion, risk reduction behavior, telemedicine

## Abstract

**Purpose:**

Primary care follow-up of women with previous GDM is often lacking. Little is known about effective approaches to improve follow-up and promote a healthy lifestyle.

**Method:**

Semi-structured individual interviews were conducted with 17 purposively sampled women with previous GDM in southern Sweden. The interviews were audio-recorded, transcribed verbatim, and qualitative content analysis was performed.

**Results:**

Three main categories were found: “a missed proactive opportunity caused by insufficient GDM follow-up”, “need for a holistic approach to health and wellbeing”, and “ambivalence toward tech-enhanced lifestyle management”. Women with previous GDM felt abandoned by healthcare after labour, since follow-up in primary care was lacking. Follow-up was viewed as proactive and requested, preferably in the form of a holistic approach to health. Motherhood and future T2DM risk were the strongest motivators for healthy lifestyle behaviours. Overall, they were cautiously optimistic regarding digital solutions in facilitating follow-up and lifestyle improvement. The capability opportunity motivation-behavior (COM-B) framework was applied, highlighting key determinants of women's ability to make lifestyle changes.

**Conclusions:**

Promoting a healthy lifestyle among women with prior GDM requires efforts at multiple levels to reduce long-term diabetes risk. Structured primary care follow-up and person-centred lifestyle support can be potentially enhanced through digital health interventions.

## Introduction

The rising global prevalence of gestational diabetes (GDM) and its association with increased risk of type-2 diabetes mellitus (T2DM), as well as cardiovascular disease later in life, is a marker of future chronic disease risk in women and a public health concern (Kramer et al., 2019; Vounzoulaki et al., 2020). GDM affects approximately 14% of all pregnancies worldwide, although reported rates vary considerably depending on screening strategies and diagnostic criteria. In Sweden, the prevalence increased from 2.6 to 6.6% when implementing WHO-2013 GDM diagnostic criteria (de Brun et al., 2024; Wang et al., 2022). Women with a history of GDM have up to a tenfold increased risk of developing T2DM, with nearly half receiving a diagnosis within the following decade (Vounzoulaki et al., 2020). Preventive measures, such as regular exercise and a healthy diet, are known to reduce the risk of developing T2DM (Aroda et al., 2015; Ratner et al., 2008). During pregnancy, women with GDM are motivated to maintain a healthy lifestyle to obtain glycaemic control, thereby reducing the risk of complications, especially for their unborn child. However, previous studies have shown that they often relapse into unhealthy habits after delivery, and while women with a history of GDM recognize the association between GDM and T2DM, they are not more likely to make healthy lifestyle changes than those with normoglycaemic pregnancies (Dennison et al., 2019; Nielsen et al., 2014). Studies have also documented the difficulty of balancing motherhood with their own health and well-being (Jenkinson et al., 2025). Furthermore, postpartum follow-up, screening, and sustained support for lifestyle change are essential for encouraging women to continue attempting to change their behaviour (Muhwava et al., 2019). Lifestyle interventions may prevent the progression to T2DM by improving weight, glucose regulation, and healthy behaviours, as concluded in systematic reviews of previous randomized controlled trials (RCTs) (Goveia et al., 2018; Hewage et al., 2020; Huang et al., 2022). To understand how and why a behaviour occurs, the COM-B model is a widely used framework that explains behaviour as the result of Capability (the individual's knowledge, skills and ability to perform the behaviour), Opportunity (external factors that make the behaviour possible or prompt it), and Motivation (internal processes that influence decision-making and direct behaviour) (Michie et al., 2011). Since there is a so-called window of opportunity to prevent T2DM as well as to avoid or delay diabetes complications, several postnatal guidelines such as the American Diabetes Association and NICE in the UK recommend diabetes screening for women with previous GDM (American Diabetes Association, 2025; National Institute for Health & Care Excellence, 2020; Ohene-Agyei et al., 2024). However, long-term follow-up after gestational diabetes remains suboptimal, and studies from Swedish, UK and US primary care settings have reported annual follow-up rates as low as 20%, despite recommendations for ongoing diabetes surveillance (Bernstein et al., 2017; Björk Javanshiri et al., 2023; McGovern et al., 2014).

Consequently, there is a need for investigating potential approaches to improve follow-up as well as interventions grounded in behaviour change theory and directed by primary care to support a healthy postnatal lifestyle among women with previous GDM to reduce the risk of future T2DM. However, limited primary care resources in relation to a wide healthcare assignment highlight the importance of exploring whether technology-based solutions may offer a complementary approach to conventional follow-up care, with the potential of providing information, facilitating communication with healthcare, and promoting healthy lifestyle behaviours (McMillan et al., 2018).

Previous European studies have explored women's experience of GDM postpartum care and highlighted challenges related to follow-up, limited awareness of future type 2 diabetes risk, and difficulties in maintaining lifestyle changes after a pregnancy complicated by GDM (Dennison et al., 2020; Svensson et al., 2018; Toft et al., 2022). However, a deeper understanding of primary care's role and health-promoting strategies is needed, particularly given its central position in preventive care. Furthermore, despite differences in healthcare organization and follow-up practices across healthcare systems, evidence from a Swedish primary care context is lacking. This study addresses this knowledge gap by exploring women's experiences of postpartum follow-up within Swedish primary care, lifestyle prevention, and their views on the use of technology to facilitate these efforts, using the COM-B model as a theoretical framework.

## Materials and methods

### Aim, design, and setting

In Sweden, screening practices for GDM vary across regions. To explore the experiences of women with a recent history of GDM regarding follow-up in primary care and lifestyle prevention, we conducted a qualitative study in primary care settings in Skåne County, southern Sweden, where nearly all pregnant women undergo GDM screening as part of routine antenatal care. Women diagnosed with GDM are referred to specialist antenatal care, which collaborates closely with endocrinologists in the management of GDM during pregnancy. After childbirth, women with GDM are referred to primary care for long-term follow-up (Berntorp & Strevens, 2023). We also aimed to explore ways to improve follow-up attendance and adherence to preventive recommendations, as well as to examine the potential role of technology in supporting this. The study adhered to the Consolidated Criteria for Reporting Qualitative Research (COREQ).

### Data sources and sampling

The study population was purposively selected from women diagnosed with GDM (ICD O24.4 and O24.4X International Classification of Diseases, ICD-10) at the Endocrinology Department, Skåne University Hospital in Malmö and Lund during 2021–2022 (*N* = 784). Women diagnosed with manifest diabetes mellitus, who had relocated from the geographic area, could not communicate in Swedish, or had experienced another pregnancy or childbirth during the study period were excluded. Eligible women, approximately 20 at a time, were invited by postal mail. The letter contained information about the study and an offer to participate in face-to-face interviews at a designated primary health care centre. If attending in person was challenging, participants were also offered the option to participate digitally via Zoom video platform or by phone. Different interviewing methods were offered intentionally to facilitate recruitment. Purposive sampling was used to obtain maximum variation. A semi-structured interview guide was developed based on different thematic areas of interest. The following thematic areas were sought:

- Overall experience of follow-up in primary care and the care provided, focusing on barriers and facilitators for attending regular follow-up.
- Views on how the follow-up should be conducted in primary care to improve compliance.
- Overall experiences of lifestyle interventions and views on how primary care can best facilitate motivation and support in adopting lifestyle changes.
- Overall experiences of digital tools used in health care and views on their potential in facilitating lifestyle changes and easing follow-up in primary care.

Eligible women were contacted by phone two weeks after the invitations were sent to provide additional information and address any questions they might have. Written or verbal consent was collected before the interviews, which took place within four weeks after the initial contact (between September and December 2023) and were audio-recorded and transcribed pseudonymously. Interviews were conducted until further interviews did not render any more information, which was perceived after 17 of them were conducted. For more detailed information on data sampling, recruitment, and interview procedure, see Björk Javanshiri et al. (2025).

### Analyzes

A qualitative content analysis approach, as described by Graneheim and Lundman, was used to analyze the data (13, 14). Transcribed interviews were read several times. For each interview, meaning-bearing units were identified, condensed, and then abstracted to codes by all involved authors. The codes were collectively synthesized into nine sub-categories and three categories that were discussed and interpreted by the research team until agreement was reached. The COM-B model was applied after the initial inductive analysis as a theoretical lens, rather than as a prior coding framework.

### Ethics

Ethical approval was obtained from the Swedish Ethical Review Authority (Dnr 2022–07173-01) before the study was conducted. The study adhered to the Declaration of Helsinki principles. Participants received an information letter describing the research, its voluntariness, and the possibility of withdrawing their participation at any time. Informed consent, written and/or verbal, was acquired before the interviews.

### Researcher characteristics, preunderstanding, and reflexivity

The first author (ABJ), at the time doing her residency in general practice, with experience working in a midwifery clinic, was responsible for data collection and interviews. Data analysis was performed by all involved co-authors, with the senior authors SC and SM involved from the commencement of the study, while PN joined later as an external reviewer. SC and SM are both female specialists in family medicine and associate professors; PN is a male general practice registered nurse with a PhD at the time, all with experience in qualitative research. None of the authors had any medical responsibility for the included study participants.

## Results

**Figure 1.**
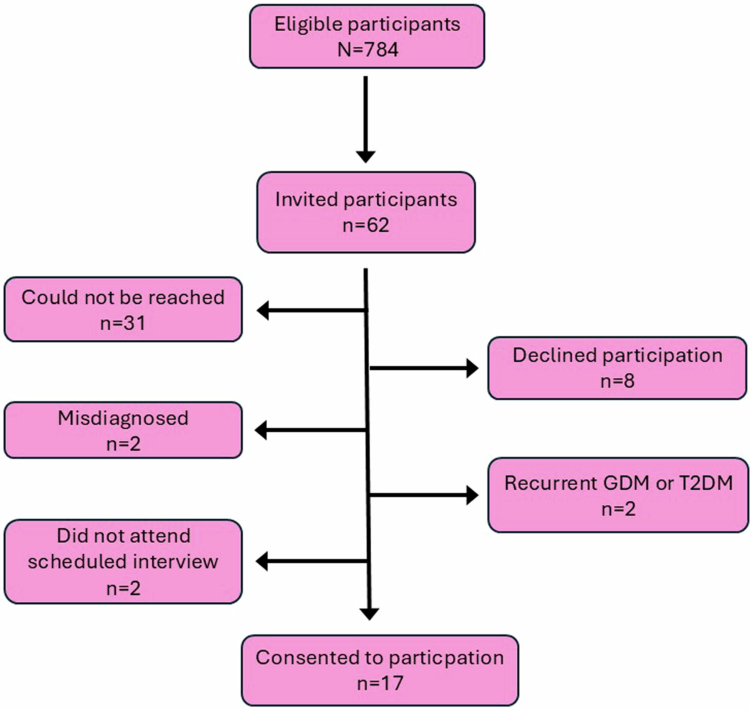
Flowchart of the study population.

A flowchart of the study population is presented in Figure 1. Seventeen interviews were conducted, which lasted 37 minutes on average.

The participants had a median age of 32 years, between one and two children, and a median self-reported body mass index of 30.1. For further details about demographics, see Table I.

**Table I.** Demographics of the study population (*N* = 17).

Characteristics	Median (range)	*n* (%)
Body mass index (BMI) kg/m^2^	30.1 (21.2–37.8)	
Age, years	32 (26–43)	
No. of children	1 (1–2)	
Ethnic background	European	1 (5)
	Non-European	2 (12)
	Sweden	14 (82)
Education	University	12 (71)
	Secondary school	4 (24)
	Elementary school	1 (5)
Smoking	Current smoker	0 (0)
	Former smoker	5 (29)
	Never smoker	12 (71)

The analysis resulted in nine subcategories (in italics), grouped into three categories labelled “a missed proactive opportunity caused by insufficient GDM follow-up”, “need for a holistic approach to health and well-being”, and “ambivalence toward tech-enhanced lifestyle management”, see Table II.

**Table II.** Overview of the qualitative content analysis results.

Categories	Subcategories
A missed proactive opportunity caused by insufficient GDM follow-up	Experience of primary care follow-up Independent follow-up Views on follow-up
Need for a holistic approach to health and well-being	Future risk of type 2 diabetes Everyday family life and motherhood Healthy eating challenges Physical activity determinants
Ambivalence toward tech-enhanced lifestyle management	Follow-up facilitated by technology Technology as a motivator for a healthy lifestyle

### A missed proactive opportunity caused by insufficient GDM follow-up

This category included the following subcategories: *experience of primary care follow-up*, *independent follow-up,* and *views on follow-up*.

The majority lacked *experience of primary care follow-up* postpartum; the abandonment was illustrated by one participant as:

‘*I gave birth, and then I disappeared*.’ (Participant 10)

Only two out of 17 participants were followed up in primary care. They perceived the follow-up as positive, reassuring them that they were healthy and on the right track regarding preventive measures. They both had their blood glucose checked, but just one had received care in line with national guidelines (including blood pressure control, lifestyle counselling, and planning for yearly follow-up). Another two participants were scheduled for follow-up, but had not yet attended it. Those who did not receive any follow-up were unsure about how future follow-ups should be conducted in primary care or were uninformed about it.

‘*So, I haven’t had any follow-up. I don’t know if I should, I think that you were supposed to after one year. But I haven’t…I don’t know if I should schedule an appointment myself, but no one has contacted me about it*.’ (Participant 6)

Participants expressed that they would appreciate and take part in follow-up in primary care, if offered. Some were unaware of lifelong follow-up in primary care being recommended. A few expressed that they, of course, would contact primary care first-hand if needed, including in case of symptoms of T2DM.

Many reported that the uncertainty regarding follow-up increased anxiety about having T2DM without knowing, which led to several participants checking their blood glucose levels themselves as one participant reported:

‘*Just because I’ve been a bit worried, like now I’ve been really thirsty and peed a lot and yes, perhaps I should check for a few days.’* (Participant 2)

This gave a sense of control to a certain degree for some, but at the same time, hesitation about how to interpret the result was expressed. It was also viewed as somewhat unreasonable to be involuntarily forced to adhere to the *independent follow-up* when health care should take that responsibility.

Plenty of *views on follow-up* and how it should be conducted in primary care were expressed. The majority felt that regular follow-up and screening for T2DM in primary care was important for their health and something that they wanted to participate in. Compliance with follow-up and screening was viewed as being proactive to prevent T2DM.

‘*It would be good with an annual check, or every other year, or something like that. To follow up and see that things are going in the right direction, or that they aren’t going in the wrong direction anyway.*’ (Participant 5)

Regarding the content of follow-up, most requested a blood glucose check and expressed that they would appreciate an overall health check. In a broader sense, this would include further blood tests, if necessary, as well as a conversation about mental well-being, lifestyle counselling, and how to manage their new life as a parent. Some expressed that they would like glucose monitoring for a few days instead of an oral glucose tolerance test (OGTT). Many thought that OGTT was repulsive and time-consuming, and expressed hesitation about OGTT as a screening method and wanted to avoid it, while others thought it to be the most reliable method. A request for follow-up with a dietitian or physiotherapist was also reported, and most were positive when this was suggested. Some reported that a dietitian could support maintaining or implementing a healthy diet as a parent, while others thought that they were well-informed and felt that it would be unnecessary, as illustrated by the following quotes:

‘*…if I had such a contact that was kind of positive and supporting and maybe helped with ideas to get in… get in the routine that I had during pregnancy when I kept on good track. To sort of bring it into life with a toddler that doesn’t eat everything*.’ (Participant 10).

‘*No, because I feel that I’ve received a lot of support, and I actually know what is required of me, I know what I should do, like I know how I should live*.’ (Participant 14)

Some expressed that they would appreciate a physiotherapist to start training postpartum, especially to strengthen the abdominal and pelvic area. They did not report that a physiotherapist could support them in sustaining or initiating working out, but a few expressed that they would appreciate some kind of support from healthcare to exercise, for example, group training.

Opinions on the timing of follow-up varied; some thought that one year after delivery was appropriate since it takes time to adjust to life as a mother, while others felt that they worried about T2DM and therefore wished for an earlier check-up.

### Need for a holistic approach to health and wellbeing

This category covered *future risk of type 2 diabetes*, *everyday family life and motherhood, healthy eating challenges*, and *physical activity determinants*.

Many found it difficult to sustain their healthy lifestyle behaviours after pregnancy. Some participants reported that the awareness of *future T2DM risk* was a strong motivator for maintaining healthy lifestyles and perceived that they could minimize this risk with a healthy diet and regular exercise. Others were aware of the risk, but it did not motivate them to make any lifestyle changes. They hoped to postpone the necessary healthy lifestyle adjustments, making the effort after being diagnosed with T2DM instead.

*Everyday family life and motherhood* could function as both a facilitator and a barrier to a healthy lifestyle. Being a mother facilitated increased movement, running after their children, and participating in their play. The fact that many babies prefer to sleep in the stroller/pram could also mean daily walks. Some reported that their own health felt prioritized as mothers since they wanted to have the energy they needed to take care of their children, and to live longer, healthier lives for the sake of their children, as well as being a good role model.

‘*And I notice that they want to include me in more of what they do. ‘Come on Mom! Come on Mom, let´s run, let´s run!’ Yes, and then you want to be able to cope and keep up. So…now I do it for the sake of the children*.’ (Participant 11)

Participants also expressed that it might be difficult to prioritize oneself as a mother, especially in the beginning, and an expectation that this will get easier as the children grow older. Some excused their bad habits by being temporary and thought that healthy lifestyles were important in the long run, therefore hoping to establish this later. Women felt that they made conscious choices, such as biking to work instead of using the car. On the other hand, others reported competing demands on time as a major issue for healthy living. Participants thought that it was challenging to sustain healthy habits since this disrupted their everyday lives, managing their work as well as being an attentive parent and partner.

‘*Yes, it’s a whole lifestyle that changes. You really must rethink, and for many, it’s quite difficult as well. You must take care of yourself, at the same time you have your child to take care of, and then there’s lack of sleep, and then there’s your partner, and then there’s your work and everything*.’ (Participant 9)

Motivators for healthy eating differed during and after pregnancy. During pregnancy, GDM motivated a healthy diet to minimize the risk of complications, especially with the expected child in mind. During pregnancy, many reported that they tried to keep a diet low in carbohydrates and high in whole grains. A few women even reported losing weight during pregnancy. The experience of GDM and the dietary advice given were maintained to varying degrees after childbirth; several found *healthy eating challenging*. Postnatally, some reported that healthy dietary choices were made automatically, while others expressed that they fell back to less healthy diet habits and stated laziness and lack of time as possible reasons for this, as expressed by one participant:

‘*Yes, I actually know what to do, but it’s so much easier…it’s just laziness, it’s so much easier to do what you’ve always done. It’s much easier just to grab what’s fastest in the store and not have to think about sugar or calorie content*.’ (Participant 14)

Most were not as strict with their diet as during pregnancy since they perceived it as too challenging and stated a need for less rigid, sustainable, long-term healthy diet habits. Limited time, sleep, and energy were stated as factors that made healthy eating difficult, especially when caring for a newborn. Some stated that when you can no longer choose when to eat, it is easier to just have a quick snack, which often contains carbohydrates.

‘*In the beginning, there are quite a lot of other things that take up time, and you might not focus on yourself the way you should, and you’re glad that you get to eat anything at all sometimes, it feels like*.’ (Participant 13)

The children's preferred food could also pose a challenge to healthy eating, since it was perceived as easier to have the same food as the child and not prepare another meal.

Participants reported a wish to engage in regular exercise and stated several different *physical activity determinants*: practical reasons such as scheduling, accessibility, and cost, extrinsic motivation (exercising with family being highlighted), and intrinsic motivation. Some women preferred to exercise outside their homes, i.e., in a gym/swimming pool/dance studio. They expressed that having a time, place, or invested money made them perceive it as a commitment difficult to obtain. Others preferred exercising at home, perceiving it as easier, more accessible, and time-efficient. Many women participated in mom-baby exercise programmes as well as stroller walks postpartum. This was viewed as fun and a way to meet and socialize with other mothers. Limited time and energy were stated as barriers to regular exercise, even though several expressed a desire to improve their workout routine. Some stated that there was time to work out in the late evenings, after putting the kids to bed, but that they then felt exhausted. Intrinsic motivation was driven by the desire to do something fun or add value by being social in activities such as swimming, dancing, or group exercises. A few were hindered by automatic motivation since they perceived exercising as boring, and others by chronic illness.

### Ambivalence toward tech-enhanced lifestyle management

This category was comprised of *follow-up facilitated by technology* and *technology as a motivator for a healthy lifestyle*.

The participants had previous positive experiences of using *digital tools as facilitators* in their contact with healthcare, particularly in their interactions with the Endocrinology department during pregnancy (when they reported their glucose levels several times daily through an app). Digital contact with healthcare also increased during the COVID-19 pandemic. Several suggested the use of yearly computerized screening reminders to enhance postpartum follow-up in primary care. When asked whether they would prefer the follow-up at the primary health care centre digitally or by phone, the opinions differed. Half preferred distant contact by phone/video call, and others preferred physical contact as it was perceived as more person-centred, thorough, and trustworthy.

‘*I think it’s easier, there won’t be any misunderstandings or such, and you see one another, I think that’s important when you talk to people*.’ (Participant 2)

This also implied that you felt trust in your general practitioner (GP) or other health care personnel, which a few expressed doubts about. Even the ones who preferred contact digitally or by phone also expressed that in case of more complex matters, i.e., if something was abnormal or in case of complications, they too would prefer to visit the primary healthcare centre (PHCC). Otherwise, digital/phone contact was perceived as more time-efficient and flexible.

‘*So, a phone call would suffice, because sometimes it’s really hard to get away from work and everything. And it’s very tricky, but it’s easier to resolve things over the phone; it’s easier to leave work for five minutes to take a call than to be away for an hour, usually*.’ (Participant 9)

Several participants were cautiously optimistic about the *use of technology to motivate a healthy lifestyle* as a supplement to ordinary follow-up and screening in primary care. Especially since technology might benefit more people when resources are limited. Many thought that self-monitoring using pedometers/smartwatches particularly motivated them to do daily exercise, and that it gave them a sense of satisfaction when reaching their goals. Workout apps provided an opportunity, facilitating training at home for some of the women. A few used some kind of diet/nutrition apps, mostly for calorie counting or nutrition content and weight loss, while a few with previous eating disorders expressed a concern that those kinds of apps could be triggering. Some asked for a dietary app with weekly dinner suggestions, customized for individuals with diabetes. When asked about lifestyle advice through a text message service, it was considered as a potentially positive reminder to encourage a healthy lifestyle, although it was perceived as essential that these text messages should be individualized and the timing should be important so as not to dismiss it:

‘*I can probably see it as a positive thing, for a little motivation, if you’re stuck in everyday life. I would probably look at it like that, as a little motivation. But also, you might have it in a follow-up, yes, but we’ll send you some information, maybe about workout, or some information about food, something like that.’* (Participant 12)

‘*I would’ve felt it as nagging and just oh, I’ve to…now I have to do it, so I would rather feel a little forced to do it and then maybe don’t really want to do it, because then you don’t have…you don’t have the motivation to do it…’* (Participant 9)

A few reported that they perceived that their smartphone could sometimes steal their attention, and worried that SMS advice or apps with lifestyle advice would further contribute to this.

## Discussion

### Summary of main findings

Women with previous GDM in southern Sweden perceived that follow-up in primary care was lacking. Follow-up was requested and considered proactive, with an expressed need for a holistic approach to health. Motherhood and the future risk of T2DM were the strongest motivators for embracing healthy lifestyle behaviours. Overall, they had a cautiously optimistic attitude toward digital solutions in facilitating follow-up and supporting lifestyle change, with the potential of providing reminders, flexibility, self-monitoring, and goal tracking.

### Comparison with existing literature

There was an obvious lack of follow-up postnatally that made women feel abandoned by primary care. Overall, there was also limited clarity regarding the role of primary care in the postnatal period, and fragmented care was one reason for this. The present study was not designed to explore system-level reasons for inadequate follow-up. However, participants frequently described uncertainty regarding responsibility for postpartum follow-up, while previous research has documented shortcomings in collaboration between healthcare providers that may contribute to women being lost to postpartum follow-up (2014; Cronin et al., 2023; McCloskey et al., 2019, Nielsen et al., 2015; Sanderson et al., 2018; Sunny et al., 2020). New guidelines on follow-up of women with previous GDM have recently been implemented in the studied healthcare region, which hopefully contribute to bridging the care gap, but this study indicates that significant efforts are needed to address the current gap (Region Skåne, 2025). Another reason for insufficient follow-up might also be the stigma attached to the condition (Björk Javanshiri et al., 2025; Davidsen et al., 2022; Toft et al., 2022). An Australian qualitative study from 2025 suggested that the stigma may lead to women distancing themselves from the diagnosis, which can complicate efforts to provide preventive care, and that appropriate psychosocial support during and after pregnancy may improve women's engagement in screening and health-promoting behaviours (Jenkinson et al., 2025).

Most participants considered that follow-up was important, since it was viewed as proactive. There were different opinions on the content of the follow-up, but they all agreed on screening. They also requested a more general “health check”, emphasizing a holistic approach to their health and well-being, which is especially appropriate in a primary care setting. The prominent result of a need for a holistic approach from healthcare has also been previously suggested, e.g., that women with previous GDM want healthcare professionals to support and motivate them to continue the lifestyle changes initiated during pregnancy (Jenkinson et al., 2025; Sandsæter et al., 2019). Facilitators and barriers to screening that appeared in the current study have also been described by Cronin et al., who uncovered different paths that can be explored to improve uptake of postpartum screening. Most notable were improved education about postpartum care, a reminder system for appointments, improved continuity of care, and more convenient testing (Cronin et al., 2023). Similar to our study, a more user-friendly and convenient blood glucose test than the OGTT was also advocated in a UK systematic review of T2DM screening attendance postpartum by Dennison et al. (2020). Interestingly, guidelines in the UK, Australia, and New Zealand recommend HbA1c testing instead (Ministry of Health, 2014; Nankervis et al., 2018; National Institute for Health & Care Excellence, 2020). Evidence suggests that personalized reminder systems may be effective in improving screening uptake (Dennison et al., 2020; Nielsen et al., 2023; Sanderson et al., 2018). A Danish RCT by Nielsen et al. further examined women's perception of the electronic reminders, concluding that the design prompted feelings of co-responsibility and care from the healthcare system, supported continuity, and helped women bridge the gap between different healthcare providers (Nedergaard et al., 2023). However, evidence regarding the effectiveness of reminders remains uncertain (Huang et al., 2023).

Following a GDM pregnancy, many women intend to adopt or maintain healthy lifestyles to prevent future T2DM but experience significant challenges in doing so. The future risk of T2DM was a strong motivator for healthy lifestyle behaviours. In contrast to our study, where women were well informed about their future risk of T2DM and how to reduce that risk, several other studies have found that the lack of risk perception is a barrier to attending postnatal screening (Dennison et al., 2020; Sanderson et al., 2018). Motherhood itself, staying healthy for the sake of their children, and being a good role model, was also a facilitator of lifestyle change. That everyday family life was a dominant motivational factor for sustainable change was evident in the current study; a finding that aligns with previous research demonstrating the significance of the well-being and quality of family life (Boyd et al., 2020; Ørtenblad et al., 2021). Eating healthily and performing regular exercise were considered time-consuming and competed with other demands, which were a significant barrier to a healthy lifestyle. The challenges of prioritizing one's own health, lack of time, and time intensity accompanying motherhood as social determinants of health have also been previously described (Jenkinson et al., 2025). Studies exploring women with previous GDM in a primary care setting are relatively scarce. Toft et al found, similar to our study, that women in Norwegian primary care experience insufficient follow-up (despite GPs managing their GDM during pregnancy) and perceived future diabetes risk as a motivator for adopting healthy lifestyle behaviours (Toft et al., 2022). However, they reported a lack of support from primary care in facilitating this and requested tailored information and lifestyle counselling (Toft et al., 2022). A meta-analysis found that lifestyle intervention aimed at women with GDM initiated within three years postpartum was highly effective in reducing the risk of T2DM, with a pooled RR of 0.57 (Li et al., 2021). Interestingly, the same study found that lifestyle intervention during pregnancy was not effective at reducing the risk of postpartum diabetes. These findings support the importance of primary care follow-up and preventive measures to promote a healthy lifestyle among this patient group.

Overall, women with previous GDM had a positive attitude toward digital solutions in facilitating follow-up and supporting lifestyle change. They proposed electronic or SMS-based screening reminders alongside telephone follow-up for patients who preferred remote consultations. Participants in the current study expressed that the use of technology provided flexibility and personalization with the possibility of including health information, tracking, and goal setting. In particular, participants expressed the need for an app that provides meal suggestions for individuals with diabetes. Evidence from several RCTs evaluating different lifestyle interventions (i.e., mHealth, health-promoting text messages, group education programme, family-based health promotion) showed mixed results, with some studies reporting challenges in achieving lifestyle changes among women with previous GDM, while others found limited effect on weight, physical activity and dietary goals (Cheung et al., 2024; Khunti et al., 2023; Kragelund Nielsen et al., 2025; Nicklas et al., 2024; O'Reilly et al., 2016). Healthy conversation skills incorporated in maternity care could effectively support women to improve their diets and physical activity levels during pregnancy and might also have a place in postpartum care (Lawrence et al., 2020). An app based on the COM-B model has been developed to promote physical activity among women with GDM and showed promising results in a feasibility study (2024, Smith et al., 2022). To conclude, interventions should be tailored to meet patient needs and may include combinations of strategies (Dineen-Griffin et al., 2019).

### Current findings in the light of the capability opportunity motivation framework

**Figure 2.**
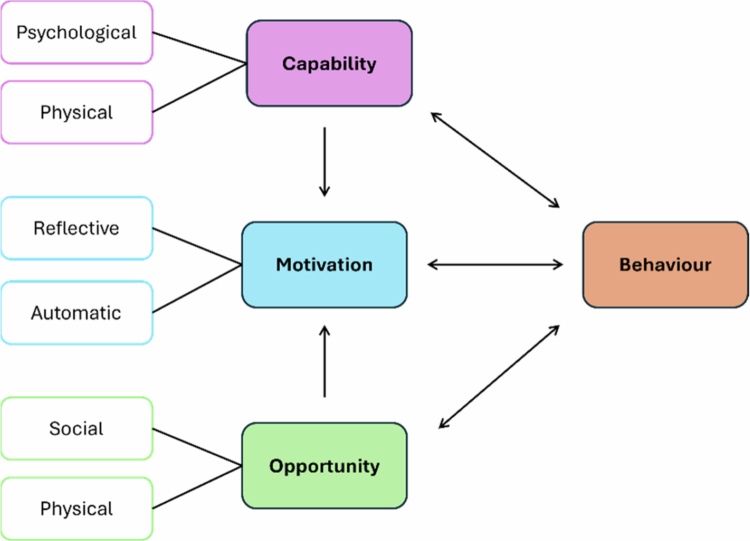
The Capability Opportunity Motivation framework for behaviour change as described by Michie et al. (2011).

Key determinants of women's ability to adopt lifestyle changes were identified and examined through the lens of the COM-B framework, see Figure 2. Women had the *psychological capability* to change their behaviour. They felt that they had the knowledge, skills, and personal desire to do so, but their new role as mothers challenged this. A few lacked *physical capabilities,* especially in the immediate postpartum period. *Opportunity* opposed a greater challenge, since their opportunities to make desired lifestyle changes were limited to their *physical environment,* such as competing demands on time and access to exercise facilities. They expressed ways to overcome these obstacles by incorporating exercise into their everyday, e.g., stroller/pram walks or mother-baby exercise. Parental leave also provided them with more time, at least temporarily. *Social opportunities* were described as encouragement from supportive partners and children, exercising together with family, and a healthy lifestyle was also perceived as being encouraged by the wider community. Taking care of their health and, by extension, the health of their children was an important *automatic motivation* as well as being a good role model. Smartwatches were stated as *reflective motivation* as they provided self-monitoring and conscious goal tracking. Participants often had reflective processes in terms of plans for working out and eating healthily, which were sometimes disrupted by *automatic processes*, feelings of procrastination, and impulses to do the opposite. In this study, we identified several aspects that are possible to address to support women with previous GDM in lifestyle prevention. First, increased awareness among GPs and other healthcare professionals could increase women's capability and motivation. Capability may be enhanced through knowledge and personalized information about future diabetes risk and preventive strategies, while motivation may be strengthened through risk communication, motivational support, and regular reminders integrated into routine follow-up care. Second, improved follow-up could also contribute partly to environmental opportunity to change. However, the possibility of providing opportunity, access to a healthy diet, and regular exercise is, of course, a greater task for policymakers and legislators. Third, technology might positively affect all parts of the COM-B model, making it a promising means for future health-promoting interventions.

Effective behavioural change interventions are central to promoting healthy lifestyles and reducing the risk of T2DM and CVD among women with a history of GDM. We found several aspects in the COM-B model that can facilitate or impede behaviour change, which has also been described previously in a low/middle-income setting (Muhwava et al., 2019). Boyd et al. investigated the utility of the COM-B model in identifying aspects of maintaining a healthy postnatal lifestyle following a diagnosis of GDM and found, like the current study, that factors related to the individual, family life, community, and health care provision all affected behaviour change. They suggested that future work should consider family-level intervention as well as using a population- and community-level approach (Boyd et al., 2020).

### Strengths and limitations

Trustworthiness was addressed through strategies targeting credibility, dependability, confirmability, and transferability. A semi-structured interview guide was thoroughly developed before the first pilot interviews and then adjusted accordingly, which contributes to the study's credibility and dependability. Another strength is the variation in the sample (participants of different ages, habitation, parity, and ethnicity), which contributed to thick description and transferability. Continuous summaries during the interviews were used to confirm accuracy as part of member checking, enhancing credibility. Field notes were taken, as well as a reflexive journal being kept. All authors contributed to the data analysis, and any discrepancies were discussed as part of investigator triangulation, with one senior author joining at a later stage, thereby increasing the dependability and credibility of the study. These strengths contribute to the trustworthiness of the study. However, as is usually the case with qualitative research, the results cannot be generalized but may be transferable to similar contexts. There is also a risk of selection, recall, and social desirability bias. All authors work in primary care, which influences our preunderstanding, although the first author (ABJ) has experience working at a midwifery clinic and collaborating with prenatal healthcare at the hospital. There is also the possibility of things getting lost in translation, since the interviews were conducted in Swedish and then translated to English by a native speaking translator; however, this was done after the coding. Furthermore, we did not apply the COM-B model in the coding process; instead, it was applied afterwards as a theoretical framework to further discuss our findings and illustrate behaviour change.

### Implications for future research and clinical practice

A major challenge for healthcare providers in supporting women with a history of GDM to change unhealthy lifestyle behaviours lies in the diversity of their individual needs and preferences. To be successful, interventions must be grounded in behaviour-changing theory and tailored to their needs. In this regard, digital technology holds considerable potential. However, technology is unlikely to replace the need for face-to-face interaction with a GP and a holistic approach to health and well-being. The findings in our study also clearly indicate that some determinants lie beyond the responsibility of healthcare, highlighting the need for broader public health interventions.

## Conclusions

By integrating a descriptive qualitative approach with the COM-B framework, this study provides valuable insight into women's experiences of postnatal follow-up after GDM, the feasibility of lifestyle changes, and the possibility of future digital health interventions in a Swedish primary care setting. Our findings highlight that promoting and supporting a healthy lifestyle among women with prior GDM requires efforts at multiple levels, including the individual, family, and healthcare system. To promote long-term lifestyle change, interventions would benefit from addressing the COM-B model. However, further research is required to assess the impact of lifestyle interventions on reducing diabetes risk and cardiovascular outcomes, as well as determining their cost and long-term effectiveness.

## Supplementary Material

Supplementary Material COREQ_Checklist.pdf

## Data Availability

The data that support the findings of this study are not publicly available due to ethical and privacy restrictions, as the data consist of qualitative interviews that could potentially identify participants. Pseudonymized data may be available from the corresponding author upon reasonable request.
